# WheatLFANet: in-field detection and counting of wheat heads with high-real-time global regression network

**DOI:** 10.1186/s13007-023-01079-x

**Published:** 2023-10-04

**Authors:** Jianxiong Ye, Zhenghong Yu, Yangxu Wang, Dunlu Lu, Huabing Zhou

**Affiliations:** 1 College of Robotics, Guangdong Polytechnic of Science and Technology, Zhuhai, Guangdong China; 2 Hubei Key Laboratory of Intelligent Robot, Wuhan Institute of Technology, Wuhan, China

**Keywords:** Wheat heads, Detection and counting, Practicality, High-real-time, Neural network

## Abstract

**Background:**

Detection and counting of wheat heads are of crucial importance in the field of plant science, as they can be used for crop field management, yield prediction, and phenotype analysis. With the widespread application of computer vision technology in plant science, monitoring of automated high-throughput plant phenotyping platforms has become possible. Currently, many innovative methods and new technologies have been proposed that have made significant progress in the accuracy and robustness of wheat head recognition. Nevertheless, these methods are often built on high-performance computing devices and lack practicality. In resource-limited situations, these methods may not be effectively applied and deployed, thereby failing to meet the needs of practical applications.

**Results:**

In our recent research on maize tassels, we proposed TasselLFANet, the most advanced neural network for detecting and counting maize tassels. Building on this work, we have now developed a high-real-time lightweight neural network called WheatLFANet for wheat head detection. WheatLFANet features a more compact encoder-decoder structure and an effective multi-dimensional information mapping fusion strategy, allowing it to run efficiently on low-end devices while maintaining high accuracy and practicality. According to the evaluation report on the global wheat head detection dataset, WheatLFANet outperforms other state-of-the-art methods with an average precision AP of 0.900 and an R^2^ value of 0.949 between predicted values and ground truth values. Moreover, it runs significantly faster than all other methods by an order of magnitude (TasselLFANet: FPS: 61).

**Conclusions:**

Extensive experiments have shown that WheatLFANet exhibits better generalization ability than other state-of-the-art methods, and achieved a speed increase of an order of magnitude while maintaining accuracy. The success of this study demonstrates the feasibility of achieving real-time, lightweight detection of wheat heads on low-end devices, and also indicates the usefulness of simple yet powerful neural network designs.

## Introduction

As one of the world's most important crops, wheat plays a critical role in global agriculture and is essential to human food supply [1–3]. With the increasing global population, wheat yield prediction has become an indispensable part of agricultural production, providing necessary reference for field management and agricultural decision-making.

Meanwhile, with the continuous development of computer vision technology, the significance of using object detection methods to identify and count wheat heads has become increasingly prominent [4]. This technology can not only monitor crop growth, but also accurately estimate wheat yield and help analyze plant phenotype characteristics, contributing to the study of wheat growth patterns and genetic traits. Therefore, the research on object detection methods for wheat is of great theoretical and practical significance [5, 6].

In recent years, Convolutional Neural Network (CNN) [7, 8] as a representative model in deep learning, has been widely used in object detection tasks due to its excellent performance in processing image and video data. The design of CNN is inspired by the working principle of the biological visual system, which achieves tasks such as image classification, object detection, and semantic segmentation by learning features within the receptive field [9–11]. In the realm of detecting and counting wheat heads, researchers have explored many other methods for detecting and counting wheat heads. Among them, the You only look once version 3 (Yolov3)-based object detection algorithm has achieved good results in wheat head detection [12], while the Faster Region-based Convolutional Neural Network (Faster R-CNN) algorithm has been applied to particle counting of wheat head [13]. Moreover, some researchers have also proposed traditional methods based on image processing and computer vision, such as morphology-based wheat head detection [14] and color segmentation-based wheat head counting methods [15]. In the field of wheat head analysis, there are also many other related studies. For example, some researchers have used infrared images to classify wheat varieties [16], while others have explored the use of laser radar technology to achieve real-time monitoring of wheat growth [17]. Furthermore, some researchers have proposed computer vision and machine learning-based methods for wheat yield estimation [18, 19] and farmland monitoring [20], providing strong support for the digital transformation of the wheat industry and agricultural modernization.

**Fig. 1:**
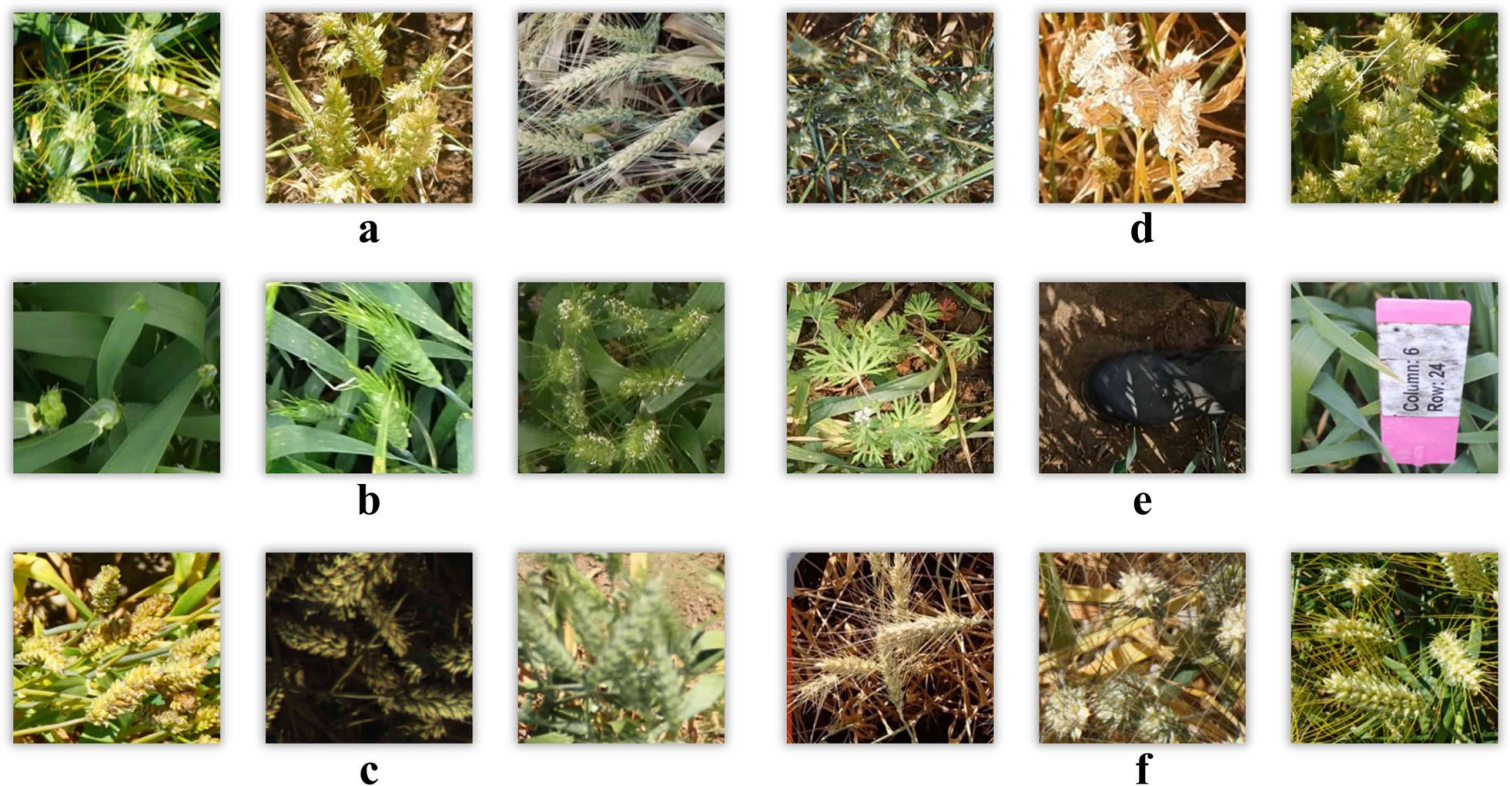
Vision challenges and difficulties in automated recognition of wheat heads. **a**. Variations in appearance due to varietal differences in different regions, **b**. Texture differences resulting from different growth stages, **c**. Changes in illumination due to varying weather conditions, **d**. Dense distribution and significant occlusion caused by precision farming, **e**. Diversity and induced visual patterns due to complex backgrounds, **f**. Posture changes caused by wind, imaging angles, and perspective differences

Encouragingly, in 2020–2021, Lowe et al. [21] released two new large-scale wheat head datasets—Global Wheat Head Detection 2020/2021 (GWHD_2020) [21] and (GWHD_2021) [22], and the research direction of wheat head detection algorithms has gradually received attention and support. However, due to the complexity of the agricultural environment and the diversity of wheat heads, as shown in Fig. 1, this dataset still poses challenges and difficulties for algorithm recognition, which can largely be attributed to:

- Variety differences and growth environment: variations in wheat species and growth environments frequently result in substantial differences in appearance between distinct wheat head images, thereby presenting a more complex challenge for recognition algorithms.
- Variations in Growth Stages: Given variations in growth stages, the texture patterns also undergo fundamental changes.
- Lighting changes: different lighting conditions at various times and weather conditions can significantly impact the appearance of wheat heads, thus making recognition more challenging.
- Overlap and intersection: wheat heads frequently intersect and overlap, which poses a challenge for detection algorithms to accurately distinguish them.
- Complex Backgrounds: The presence of intricate and cluttered backgrounds significantly compounds the challenge for algorithm recognition.
- Angle and scale changes: changes in the camera’s position and shooting scale can also alter the appearance of wheat heads in the image, increasing the difficulty of recognition.

In the research of wheat head detection algorithm, researchers are facing various challenges and difficulties. In order to improve the accuracy and robustness of the algorithm, many innovative methods and techniques have been proposed. For example, Wang et al. [23] introduced an enhanced approach for wheat head counting by utilizing an improved EffificientDet-D0 object detection model. This method specifically addresses the challenge of occlusion in wheat head detection. To simulate occlusion scenarios encountered in real wheat images, the researchers employed the image enhancement technique of d Random-Cutout, which selectively applied rectangles to mimic occluded regions. Additionally, Sun et al. [24] used an improved wheat head counting network (WHCnet) that enhances the detection and localization accuracy of dense wheat heads by optimizing the resampling strategy in the high threshold stage. Li et al. [25] employed the R-CNN approach for wheat head detection, counting, and analysis, achieving high recognition accuracy. However, this method exhibits slow detection speed and is unable to meet certain requirements. Similarly, Carion et al. [26] proposed a DEtection TRansformer DETR algorithm based on Transformer for object detection of wheat heads, which has better interpretability and efficiency compared to traditional detection methods and achieved good results. What’s more, Zhou et al. [27] also used a wheat head detection method based on Transformer architecture, which achieved high accuracy and robustness in complex agricultural environments. Nevertheless, due to the use of the Transformer network structure, a large amount of training data and computational resources are required to achieve good performance [28], which may limit its practical application.

Overall, with the continuous efforts of machine learning experts, the accuracy and robustness of wheat head recognition have made significant progress. Yet, these advances are often based on high-performance computing resources and environments, and in practical applications, the real-time lightweight problem of the algorithm is a key challenge. Specifically, existing algorithms often require a lot of computing resources and time to train and optimize the model, and may have requirements for specific hardware platforms and software environments, making it difficult for them to achieve good performance in different deployment environments. Moreover, for some rural areas and open environments, device resources may be very limited, and high-performance algorithms are often difficult to deploy and use.

Regarding the above issues, we continued to search for studies that were more likely to achieve real-time performance. Our attention was focused on the Yolo algorithm because it has an impressive balance of accuracy and speed in the field of object detection. In our research, we found that Yang et al. [29] and Gong et al. [30] used an improved Yolov4 algorithm, while Zang et al. [31] used an improved Yolov5 algorithm. These improvements mainly included using attention mechanisms to improve detection accuracy and using lightweight models to improve algorithm real-time performance and deployability. Interestingly, these studies had a small number of test samples and even used very unreasonable training-to-testing ratios, which may not cover all wheat varieties and growth environments. Therefore, these results may have some randomness and dramatization. Besides that, Khaki et al. [19] designed a lightweight wheat spike detection and counting network WheatNet using MobileNetv2 as the backbone. Based on the single-stage network framework, Sun et al. [67] proposed a lightweight WDN model for wheat heading detection and counting. Nonetheless, their generalization ability is likely to be limited, and they may have limitations in capturing complex patterns and expressing complex relationships in the data.

In the field of agriculture, we previously studied wheat heads as a crop in early visual applications. With the development of computer vision technology, we gradually shifted towards using object detection methods for automated identification and counting of crops. In this process, we discovered some previous studies, such as a wheat field automatic detection method based on image processing [32], which can automatically count and estimate the number of wheat heads and help analyze the growth patterns and genetic characteristics of wheat. These early studies laid the foundation for our later research on maize tassels and promoted the continuous development of visual research in the agricultural field [33–35]. Not long ago, we achieved new results in our maize tassel research by proposing a globally-regressed object detection framework neural network called Tassel Lightweight Feature Aggregation Network (TasselLFANet) [36] and achieved SOTA performance in field maize tassel counting applications. It is worth mentioning that in this work, we also tried various object detection methods and showed that the current state-of-the-art detection methods perform well in similar plant counting applications. Based on this, we used TasselLFANet as a baseline and conducted experiments on wheat heads, but we found that TasselLFANet still has the following limitations in practical applications:

- Long training time: the model takes a long time to converge to a suitable accuracy during training.
- Large number of parameters: the model has a large number of parameters, which increases the computational cost.
- High memory usage: the model requires a large amount of memory to store parameters and intermediate results.
- Long data processing time: the model requires a long time for data preprocessing.

Therefore, based on the overall network architecture of TasselLFANet, we constructed a more lightweight Wheat Lightweight Feature Aggregation Network (WheatLFANet) neural network while maintaining high accuracy. Through careful design, this neural network has a more compact encoding and decoding structure, greatly reducing the number of learning parameters, and its high-real-time lightweight nature makes it easy to deploy on mobile devices. Per the assessment report, our enhancements demonstrate substantial significance. Also, we further studied the Multi-Efficient Channel Attention (Mlt-ECA) module in previous work.

In general, this paper has three main contributions:

- We conducted a detailed review of the research on wheat head detection and found that significant progress has been made in wheat head recognition accuracy. Nevertheless, there is an urgent need to improve the application of these methods on resource-limited devices.
- Based on the state-of-the-art TasselLFANet neural network architecture, we designed a multi-dimensional mapping global regression network, WheatLFANet, which achieves high-real-time lightweight performance while maintaining accuracy. This provides a new approach for the practical application of wheat head detection under resource-constrained conditions.
- Compared to cutting-edge deep learning methods, our method has achieved an order of magnitude faster speed, outperforming some of the latest methods currently available.

## Materials and methods

### Dataset analysis

**Fig. 2:**
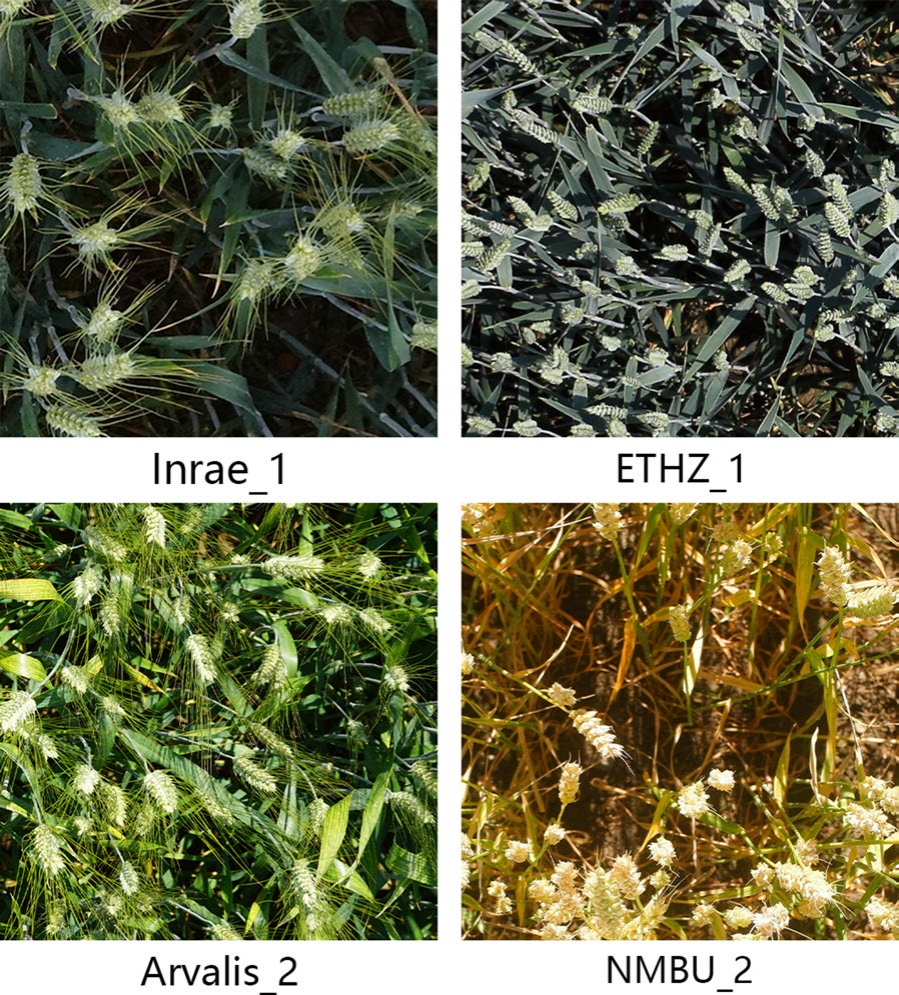
Samples of the GWHD_2021 dataset. The subscript corresponds to the source

In this work, we evaluated the performance of WheatLFANet using the Global Wheat Head Detection 2021 (GWHD_2021) [22] dataset. The GWHD_2020 [21] dataset was created in 2020 and collected 4700 RGB images with 193,634 annotated wheat heads from various platforms and 7 countries/institutions. Subsequently, an updated version, GWHD_2021, was released in the following year, which added 1722 images from 5 countries and 81,553 new wheat head instances, making the dataset larger and more diverse. As shown in Fig. 2, we present some example images.

**Fig. 3:**
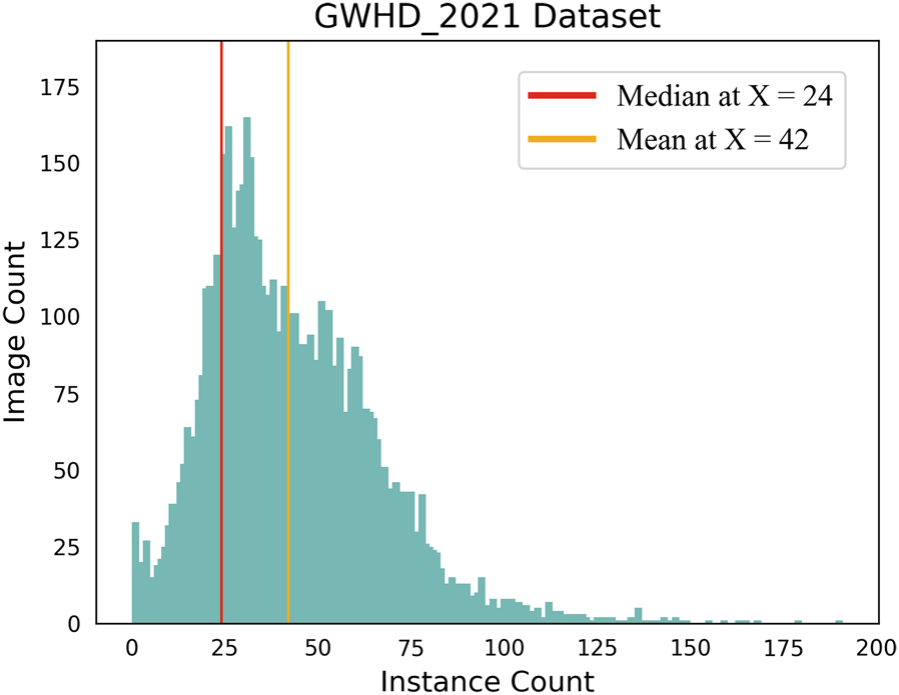
Instance distribution in GWHD_2021 dataset. The red line represents the median number of instances per image, and the yellow line represents the average number of instances per image

To further understand the distribution of the number of objects in the dataset, we counted the number of instances in each image, as shown in Fig. 3. The results showed that most images had less than 100 instances, with a median number of instances per image of 24, and an average of 42 instances per image. These data indicate that GWHD_2021 is a dataset with a large number of object instances, and there is a significant difference in the number of objects in different images. This is important for selecting appropriate object detection algorithms, adjusting hyperparameters, and evaluating model performance.

It is worth noting that in order to improve the model's generalization and anti-interference ability, making it more suitable for practical scenarios, we merged multiple varieties of wheat heads in GWHD_2021 into one category to reduce overfitting to specific varieties. This way, we can better train a model with strong generalization performance and achieve better results in practical applications. However, it should be noted that detecting multiple wheat varieties as one object will bring greater challenges. This is because different wheat varieties have significant differences in morphology and color. For example, some varieties may grow taller, have wider leaves, or darker colors, while others may be the opposite. In addition, the growth environment and growth stage of wheat also affect its appearance, such as climate conditions and soil quality, which can all have an impact on the appearance of wheat. Therefore, if multiple wheat varieties are detected as one object, the model needs to learn to recognize and adapt to their different characteristics, which inevitably increases the difficulty of model training and detection. In summary, our work aims to improve the practicality and efficiency of wheat head detection, while addressing issues such as overfitting to specific varieties, and providing better solutions for practical application scenarios.

### Design of WheatLFANet

**Fig. 4:**
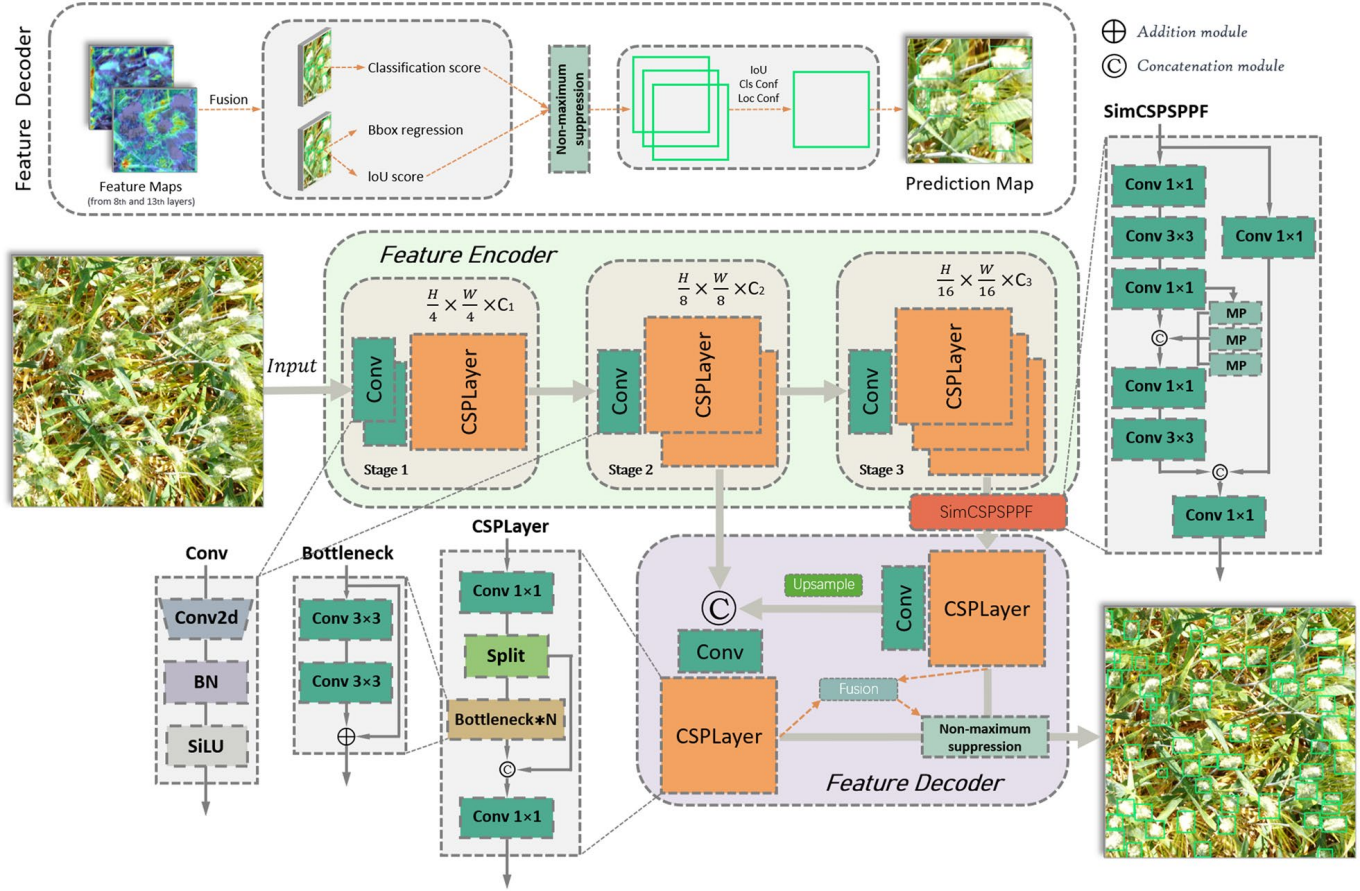
WheatLFANet global regression architecture. The output channel numbers C_1_, C_2_, and C_3_ are 32, 64, and 128, respectively, with (Conv k × k) where k is the size of the convolutional kernel. Compared to the core architecture of TasselLFANet, the downsampling method is replaced by a normal Conv layer, and the feature mapping layer is a lighter CSPLayer

TasselLFANet achieves end-to-end global regression by directly mapping image pixels to bounding box, coordinates, and classification rates. Nonetheless, in practical applications, TasselLFANet has drawbacks such as long training time, large number of parameters, high memory usage, and long data processing time. WheatLFANet aims to address these issues, following the overall architecture of TasselLFANet and creating a lightweight hybrid design that further optimizes model parameters and computational complexity, making it more efficient to run on resource-constrained devices. The overall architecture is shown in Fig. 4, and the functions and detailed structures of each module are described below.

#### Global architecture

WheatLFANet consists of two main stages: feature encoding and cross-stage fusion, with two main layers: (a) Convolution Layer (Conv); (b) Cross Stage Partial Layer (CSPLayer). Based on the overall design architecture of TasselLFANet, hierarchical features are extracted at three different scales in Stage 1, Stage 2, and Stage 3 of feature encoding, and semantic information is conveyed through multi-dimensional mapping. In the second stage, starting from the layer of Simplified Cross Stage Partial Spatial Pyramid Pooling Fast (SimCSPSPPF) [37], the size of the input image is reduced to 1/16. To deal with scale and perspective changes in the image, the output feature map is upsampled using nearest neighbor interpolation to increase the spatial dimension between cascades, obtaining a feature map with the same output size as Stage 2. This feature map is then merged with the 1/8 output of the feature encoding stage using Conv operation to map the feature maps to the same channel dimension for Concatenation, and the concatenated feature map serves as the second branch of the decoder. The feature information is remapped through CSPLayer and then the features from different layers are fused in the cross-stage fusion. Finally, the prediction layer performs convolution and nonlinear transformations on the fused feature map, and outputs the predicted box coordinates and object class.

#### Feature encoding

Given an RGB image of size $X \in R^{H \times W \times C}$ as a three-dimensional tensor, Stage 1 first processes it with two overlapping Conv layers with a stride of 2 and a kernel size of 3 × 3, producing a feature map of size $H/4 \times W/4 \times C_{1}$, which is then passed to the CSPLayer to extract local features. Stages 2 and 3 combine the same downsampling convolutional layer operation and CSPLayer to extract higher-level semantic information. This combination enables the convolutional neural network to better capture abstract features in the image, improving its perceptual ability and classification accuracy. Meanwhile, the CSPLayer further enhances feature expression by cross-channel information interaction.

The Conv layer uses 2D Convolution (Conv2d), Batch Normalization (BN) [38], and Sigmoid-Weighted Linear Unit (SiLU) [39] activation functions to enrich local representations, in order to enhance nonlinear feature mapping. Its definition can be summarized as follows:

1 $$Z_{i,j,p} = \hat{y}_{i,j,p} \cdot \sigma \left( {\hat{y}_{i,j,p} } \right) = \hat{y}_{i,j,p} \cdot \frac{1}{{1 + \exp ( - \hat{y}_{i,j,p} )}}$$

$Z$ is the output channel number, $\sigma ( \cdot )$ represents the sigmoid function, and $\hat{y}_{{{\text{i}},j,p}}$ is defined as:

2 $$\hat{y}_{i,j,p} = \frac{{y_{i,j,p} - \mu_{p} }}{{\sqrt {\sigma_{p}^{2} + \varepsilon } }}$$

$\mu_{p}$ and $\sigma_{p}$ are the mean and standard deviation of the p-th channel across all samples in the current batch, respectively. $\varepsilon$ is a small constant to avoid division by zero in the denominator, and $y_{i,j,p}$ represents the value at the i-th row, j-th column, and p-th channel of the output tensor. The equation can be defined as follows:

3 $$y_{i,j,p} = \sum\limits_{r = 0}^{k - 1} {\sum\limits_{s = 0}^{k - 1} {\sum\limits_{q = 0}^{c - 1} {W_{{r,s,q,p^{{X_{i} + r,j + s,q}} }} } } }$$

$W_{{{\text{r}},s,q,p}}$ represents the parameters of the convolution kernel, the size of the convolution kernel is $\left| {k \times k} \right|_{odd}$, and odd specifies that $k$ is odd.

As shown in Fig. 4, the number of CSPLayer modules N in Stage 2 and Stage 3 is the number of bottleneck modules. Drawing on the Efficient Layer Aggregation Network (ELAN) [40] used in TasselLFANet and the gradient flow extraction idea in CSPNet [41], the CSPLayer module was designed with a branch consisting of two Conv layers, a Split operation, and a Bottleneck block. The Conv + Split operation reduces parameter and computational complexity, and improves model generalization, helping the neural network better handle complex tasks. In the Split operation, the input tensor is divided into multiple branches or paths, and each branch undergoes separate Conv operations before being merged back together. The main branch gradient module is a residual bottleneck block, and the number of stacked modules is controlled by the parameter N. Therefore, CSPLayer can obtain richer gradient information while ensuring lightweight, thereby achieving higher accuracy and more reasonable latency. Suppose the input data is $a \in R^{{n \times c_{1} \times h \times w}}$, where $n$ represents the batch size, $c_{1}$ represents the number of input channels, and h and w represent the height and width of the input data, respectively. The output data is $A \in R^{{n \times c_{2} \times h \times w}}$. In bottleneck, the input data is first subjected to a $1 \times 1$ convolution operation to reduce the number of input channels to $c_{2} \times e$, where e is an expansion coefficient. Then, a convolution operation is performed on the first convolution result using a kernel size of $k_{1} \times k_{1}$, resulting in a set of output feature maps with a size of $c_{2} \times h \times w$. Next, a convolution operation with a kernel size of $k_{2} \times k_{2}$ is used to adjust the output channel number to $c_{2}$. Finally, the results of the first and third steps are added to obtain the final output. This operation can be described by the following equation:

4 $$A_{i,j,p} = a_{i,j,p} + \sum\limits_{q = 0}^{{n_{1} - 1}} {\sum\limits_{r = 0}^{{k_{1} - 1}} {\sum\limits_{s = 0}^{{k_{1} - 1}} {Q_{{r,s,q,p^{{a_{i} + r,j + s,q}} }} } } }$$

where $A_{{{\text{i}},j,p}}$ represents the value of the p-th channel in the i-th row and j-th column of the output tensor, $a_{i,j,p}$ represents the value of the p-th channel in the i-th row and j-th column of the input tensor, $k_{1}$ represents the kernel size of the first convolution, $n_{1} = c_{2} \times e$ represents the number of channels after the first convolution, and $Q_{r,s,q,p}$ represents the convolution kernel parameter in the first convolution operation.

#### Cross-stage fusion

The purpose of cross-stage fusion is to gather multi-dimensional mapping information, interact across different dimensions, enhance feature reuse and hierarchy, and aggregate diversified information flow. We achieve this by upsampling the output feature maps of the feature mappings again before the feature map fusion. Prior to the feature map fusion, we use the Simplified Cross Stage Partial Spatial Pyramid Pooling Fast (SimCSPSPPF) module to separate contextual information by pyramid pooling of feature maps with different receptive fields to reduce information loss and obtain richer contextual information while retaining positional information. Its structure is shown in Fig. 4, Given an input $x$ and an output $O_{p}$, the forward propagation process can be described using the following formula:

5 $$x_{1} = f^{1 \times 1} \left( {f^{3 \times 3} \left( {f^{1 \times 1} \left( x \right)} \right)} \right)$$

6 $$y_{0} = f^{1 \times 1} \left( x \right)$$

7 $$y_{1} = Maxpool\left( {x_{1} } \right)$$

8 $$y_{2} = Maxpool\left( {y_{1} } \right)$$

9 $$y_{3} = f^{3 \times 3} \left( {f^{1 \times 1} \left( {Concat\left( {\left[ {x_{1} ,y_{1} ,y_{2} ,Maxpool\left( {y_{2} } \right)} \right]} \right)} \right)} \right)$$

10 $$O_{p} = f^{1 \times 1} \left( {Concat\left( {y_{0} ,y_{3} } \right)} \right)$$

Here, $f^{k \times k}$ represents a convolution operation with a kernel size of $k \times k$, Maxpool represents Max Pooling (MP), Concat represents concatenation module, and $x_{i}$ and $y_{i}$ represent the results of non-linear transformations. The entire module consists of multiple Conv layers, pooling layers, and concatenation operations. MP operation is applied multiple times for progressive downsampling of the features, reducing the spatial dimensions of the input feature maps while preserving the most relevant information. The advantage of SimCSPSPPF is that it combines the strengths of Cross-Stage Partial Networks (CSP) [42] and Spatial Pyramid Pooling (SPP) [43]. It should be emphasized that its output represents a subset of the original features and undergoes CSPLayer processing.

Furthermore, in the final output of the feature decoding, two branches undergo fusion operations to merge the layers. The purpose of this operation is to fuse the Conv2d and BN layers into a new convolutional layer, thereby reducing the number of layers in the model and improving runtime efficiency. After this, binary cross-entropy loss is used as the supervision signal for prediction. The classification branch and box regression branch work in parallel, with the box regression branch predicting the four coordinates of each box and the object score. If the overlap between the anchor box and the ground truth box is higher than other anchor boxes, the object value is 1. Finally, the Non-Maximum Suppression (NMS) [44] algorithm is used to filter out redundant detection results from the generated predicted boxes. The intersection over Union (IoU) evaluation metric measures the overlap between two predicted boxes and compares the IoU values of the predicted boxes to determine whether they belong to the same object. IoU is calculated using the following formula:

11 $$IoU\left( {A,B} \right) = \frac{{\begin{array}{*{20}c} {Area} & {of} & {Overlap} \\ \end{array} }}{{\begin{array}{*{20}c} {Area} & {of} & {Union} \\ \end{array} }} = \frac{A \cap B}{{A \cup B}}$$

$A$ and $B$ represent two rectangular regions, ∩ denotes intersection, and ∪ denotes union.

### Loss function

The loss function describes the difference between model predictions and true labels and guides the optimization process of model parameters. A suitable loss function is crucial for the success of machine learning tasks. In the construction of the WheatLFANet network, the loss function is defined as follows.

#### Localization loss for training

Localization loss is used to evaluate the distance between the model's detected candidate boxes and the ground-truth boxes. A good bounding box regression function includes three elements: overlap area, center distance, and aspect ratio. Assuming $b_{pd}$ and $bgt$ are the center points of the predicted box and the ground-truth box, respectively:

12 $$L_{loc} = IoU - \frac{{\rho^{2} (b_{pd,} b_{gt} )}}{{c^{2} }} - \alpha v$$

$\rho$ calculates the Euclidean distance between the two centroid points, while $c$ represents the diagonal length of the minimum enclosing rectangle around the predicted box and the ground truth box. $IoU$ measures the intersection over union of the predicted box and the ground truth box. The parameter $v$ is used to quantify the similarity of aspect ratios, and $\alpha$ is its corresponding weighting factor.

#### Objective loss and classification loss

Objective loss and classification loss are loss functions based on binary cross-entropy (BCEWithLogitsLoss) and are mainly used to alleviate the impact of missing labels. Assuming the object value (label value) is $\vec{y} \in \left\{ {0,1} \right\}^{n}$ and the predicted result is $\vec{y} \in R^{n}$, the binary cross-entropy is first used as the basic loss function to calculate the error between the predicted result and the object value:

13 $$L_{bce} = - \frac{1}{n}\sum\limits_{i = 1}^{n} [ y_{i} \log \hat{y}_{i} + (1 - y_{i} )\log (1 - \hat{y}_{i} )]$$

Here, $\hat{y}_{i} = \sigma \left( {\hat{y}_{i}{\prime} } \right)$, $\sigma \left( \cdot \right)$ represents the sigmoid function. Through the Sigmoid function, the predicted result $\vec{y}$ is transformed into a probability value $\vec{p}$:

14 $$\vec{p} = \frac{1}{{1 + \exp ( - \hat{y}_{i} )}}$$

To minimize the impact of missing labels on model training, it is necessary to reduce their error. This is because the original binary cross-entropy calculation can introduce significant errors, which require adjustments to mitigate their impact on model training. Specifically, a decreasing function $\alpha \left( x \right)$ is used to reduce the error, where $x = \hat{y} - y$ is the difference between the predicted result and the object result:

15 $$\alpha \left( x \right) = 1 - \exp^{{}} \left( {\frac{x - 1}{{\alpha + \varepsilon }}} \right)$$

The $\alpha$ is a hyperparameter and $\varepsilon$ is a small value to prevent the denominator from being zero. As x gradually increases, $\alpha \left( x \right)$ approaches 1, making the error smaller and smaller. Integrating the above three parts, we obtain the loss function:

16 $$L_{jx} \left( {p,y} \right) = \frac{1}{n}\sum\limits_{i = 1}^{n} {\alpha \left( {p_{i} - y_{i} } \right)} \cdot L_{bce}$$

In the equation, n represents the number of samples, $p_{i}$ represents the predicted probability of the i-th sample, and $y_{i}$ represents the actual label. The final loss of WheatLFANet is defined as the weighted sum of localization, object, and classification losses, where the weights α, β, and γ are hyperparameters that control the relative importance of each loss:

17 $$L_{wht} = \alpha \cdot L_{loc} + \beta \cdot L_{jx}^{obj} + \gamma \cdot L_{jx}^{cls}$$

In the experiments, the values of α, β, and γ were set to 0.05, 0.7, and 0.3, respectively. The localization loss measures the difference between predicted and ground-truth bounding boxes, the object loss measures the confidence of object presence in the predicted bounding box, and the classification loss measures the accuracy of the predicted class label. The overall loss function helps to train the model to accurately detect and classify wheat heads in images.

## Experiments and discussions

In this section, we conduct a series of experiments on detection and counting tasks to validate the effectiveness of WheatLFANet. Firstly, we introduce the implementation details and evaluation metrics of WheatLFANet. Then, we conduct ablation experiments to determine the selection of core modules. Next, we compare our method with state-of-the-art methods. Finally, we validate our method on counting tasks.

### Implementation details

In this study, we used 6,387 images from the GWHD_2021 dataset, which were randomly split into training, validation, and test sets with a ratio of 7:2:1. The training set contained 4,471 images, the validation set contained 1,277 images, and the test set contained 639 images. To ensure the objectivity of the results, all methods used the same configuration for training and testing. The training device is based on Nvidia RTX 3090 (24G), Intel i9-12900 K CPU (64G), It should be noted that we did not rely on pre-trained model weights during transfer learning, in order to ensure that our model's performance reflects its true potential [45–47]. The longest side of the input image was scaled to 640 pixels, and the other side was scaled proportionally to maintain the original aspect ratio of the image. Since WheatLFANet converges quickly, the iteration was set to 100 epochs, batch size was set to 8, the learning rate was initialized to 0.01, decayed with the cosine function schedule, stochastic gradient descent was used as the optimizer with a momentum factor of 0.937 and a weight decay of $5 \times 10^{ - 4}$. In addition, all other parameters of the models used in this study were consistent with the default parameters and were not adjusted.

### Evaluation metrics

We use the following evaluation metrics to quantify the detection performance: precision (P_r_), recall (R_e_), and average precision (AP). P_r_ represents the proportion of correctly predicted objects among all predicted objects by the model, R_e_ represents the proportion of correctly predicted objects among all true objects, and AP represents the mean area under the P_r_-R_e_ curve. They are formulated as follows:

18 $$P_{r} = \frac{TP}{{TP + FP}}$$

19 $$R_{e} = \frac{TP}{{TP + FN}}$$

20 $$AP = \int_{0}^{1} {P_{r} (R_{e} )d(R_{e} )}$$

where $TP$, $FP$, and $FN$ represent the numbers of true positives, false positives, and false negatives, respectively. The evaluation metrics for counting task are as follows:

21 $$MAE = \frac{1}{N}\sum\limits_{n = 1}^{N} {\left| {G_{n} - P_{n} } \right|}$$

22 $$RMSE = \sqrt {\frac{1}{N}\sum\limits_{n = 1}^{N} {\left| {G_{n} - P_{n} } \right|}^{2} }$$

23 $$MAPE = \frac{1}{N}\sum\limits_{n = 1}^{N} {\left| {\frac{{G_{n} - P_{n} }}{{G_{n} }}} \right|} \times 100\%$$

24 $$R^{2} = 1 - \frac{{\sum\limits_{n = 1}^{N} {(G_{n} - P_{n} )^{2} } }}{{\sum\limits_{n = 1}^{N} {(\overline{G}_{n} - P_{n} )^{2} } }}$$

Here, $N$ represents the number of images, $G_{n}$ and $P_{n}$ represent the predicted and ground-truth counts in the n-th image, respectively.

### WheatLFANet key selection

The feature extraction module is a core component of deep learning models, and selecting an efficient feature extractor can help the model better understand and learn information from the data. In this section, we used ablation experiments to determine the key selections of WheatLFANet. The selected feature extractors are: RepVGG [48] with reparameterization, ConvNeXt [49] with full convolution, ShuffleNetV2 [50] as a lightweight option, and CSPDarkNet [51] known for its efficiency. To balance the model's lightweight and high-real-time requirements, we also focused on the following metrics:

- Floating point of operations (FLOPs): refers to the total number of floating-point operations executed by the model during inference, and is often used to determine the model's computational complexity. It should be emphasized that FLOPs are related to the input image size and should be clarified accordingly.
- Params: the number of model parameters is a direct measure of model complexity and an important constraint for practical deployment of the model. Models with more parameters usually require more deployment resources.
- Latency: the time required from input data entering the model to generating output results. Lower latency means faster model response time. Unlike FLOPs, inference time depends on the hardware used and the size of the input image.
- Frames per second (FPS): the number of image frames the model can process in a unit of time. Higher FPS means the model can quickly process input data and produce output results.

Please note that all experimental tests in this work were conducted on a low-end computer configuration with Nvidia GTX 1650 GPU (4G) and Intel i5-10200H CPU (8G), which has slower computational speed. Therefore, readers need to consider these limiting factors when analyzing and interpreting the experimental results. Overall, using affordable low-end devices can better extend our insights into practical applications.

As shown in Table 1, according to quantitative analysis, we found that the four extractors have similar performances in terms of precision, recall, and average precision, but there are some differences in frames per second and inference latency. Among them, ShuffleNetV2 has the highest frames per second, reaching 170 FPS, but its inference latency is similar to CSPDarknet. Overall, CSPDarknet performs relatively well in multiple aspects such as P_r_, R_e_, AP, FPS, and Latency, and its parameter count and floating-point operation count are in a better position compared to the other three extractors. This means that CSPDarknet can achieve high-precision detection at a faster speed and with fewer resources. Therefore, from a performance perspective, choosing CSPDarknet seems to be a good decision.

**Table 1:** Ablation experiments of different feature extraction modules

Method	Feature Extractor	Pr	Re	AP	FPS	Latency	Params	FLOPs
WheatLFANet	RepVGG	0.896	0.835	0.887	157	8.6 ms	1.04 M	5.65G
	ConvNeXt	0.878	0.827	0.859	90	13.3 ms	0.92 M	5.03G
	ShuffleNetV2	0.881	0.802	0.859	**170**	**8.3 ms**	**0.29 M**	**1.65G**
	CSPDarknet	**0.905**	**0.843**	**0.900**	164	8.4 ms	0.72 M	4.07G

Bold text indicates the best results

### Comparison with state of the art

To validate the effectiveness of our proposed method, we compared WheatLFANet with three state-of-the-art methods, all of which are applicable for object detection and counting in images. Respectively:

- CenterNet: proposed in [52], CenterNet is an advanced object detection framework that has gained significant attention for its exceptional performance in terms of accuracy and efficiency. It introduces a key concept called object center estimation, which accurately predicts the center location of objects in an image. This estimation, combined with a heatmap representation, allows CenterNet to achieve precise and reliable object detection. By directly predicting object centers, CenterNet eliminates the need for complex anchor generation and matching processes, leading to improved speed and simplified model architecture.
- Yolov7: proposed in [53], Yolov7 is one of the latest detectors among all known object detectors and has achieved great success in speed and accuracy within the range of 5FPS to 160FPS. It significantly improves speed and accuracy without introducing any major architectural changes. Also, its planned re-parameterization convolution and guided label assignment strategy from coarse-to-fine are referred to as "freebies" that promote better learning of the model without actually increasing the training cost. It is need to clarify that YOLOv7-tiny was selected in this study because it is specifically designed for edge architecture within the YOLOv7 series.
- EfficientDet: proposed in [54], EfficientDet balances network depth, width, and resolution to improve network performance. Specifically, the EfficientDet model uses a simple and efficient compound coefficient system to scale all dimensions of depth/width/resolution. Additionally, a novel Bi-Directional Feature Pyramid Network (BiFPN) is introduced that can effectively fuse features across scales. The model also incorporates multiple optimization techniques such as weighted feature fusion, IoU loss, and focal loss to further improve its performance.
- TasselLFANet: proposed in [36], is the state-of-the-art method for detecting and counting maize tassels in crop images. The network achieves real-time detection in natural canopy images with a large number of maize tassels, through multi-branch feature aggregation and channel-domain attention mechanisms, as well as an efficient and flexible encoder-decoder architecture. Its detection accuracy and counting performance surpass the latest batch of lightweight neural networks, and its counting accuracy is not affected by geographical changes, making it a reliable tool for maize tassel counting.

#### Results and analysis

The experimental results of the different methods on the test set are shown in Table 2. Based on qualitative results, we have the following analysis:

- P_r_, R_e_ and AP: in object detection, precision, recall, and average precision are important indicators for measuring model performance. From the data, it can be seen that Yolov7, TasselLFANet, and WheatLFANet all have precision and average precision above 0.9. By employing a dense prediction strategy, Centernet achieves a higher recall rate compared to other models, while EfficientDet's performance is relatively weak, with only 0.775 precision, 0.765 recall, and 0.804 average precision. This may be because after all wheat heads of different varieties in the GWHD_2021 dataset were covered as one category, the significant differences between different wheat head varieties were ignored, which increased the recognition difficulty. EfficientDet cannot distinguish the differences between different varieties well, which affects its accuracy and performance. In comparison, Centernet, Yolov7, TasselLFANet, and WheatLFANet have better wheat-specific capabilities.
- FPS and latency: in practical applications, object detection models need to achieve real-time performance, so frame rate and latency are very important indicators. From the data, it can be seen that WheatLFANet has the highest frame rate, which can reach 164 FPS. The frame rates of CenterNet, TasselLFANet, and Yolov7 are 44 FPS, 61 FPS, and 72 FPS, respectively, while EfficientDet's frame rate is only 19 FPS, which cannot meet the needs of real-time applications. Moreover, WheatLFANet has the lowest latency, only 8.4 ms, which means that our proposed method can complete more tasks in a very short time, thereby reducing system costs and resource consumption.
- Params and FLOPs: parameters and FLOPs are indicators for measuring model complexity and computational complexity, and they are also important factors that affect model performance and application costs. From the data, it can be seen that CenterNet has the highest number of parameters, which is 63.96 M. What’s more, Yolov7 has the second-highest number of parameters, with 6.21 M. TasselLFANet has the highest FLOPs, which is 18.72G. Relatively speaking, EfficientDet has the lowest FLOPs, which is 2.52G, but this did not give it any advantage in speed. It is worth noting that WheatLFANet has only 0.72 M parameters, which provides important basis for the model to be deployed and optimized in edge or mobile devices more easily.

**Table 2:** Detection results based on different methods. The test is based on Nvidia GTX 1650 GPU (4G)

Method	Pr	Re	AP	FPS	Latency	Params	FLOPs
CenterNet	0.808	0.890	0.880	44	25.2 ms	63.96 M	24.51G
Yolov7	0.903	0.846	0.906	72	16.6 ms	6.21 M	13.84G
EfficientDet	0.775	0.765	0.804	19	51.3 ms	3.93 M	**2.52G**
TasselLFANet	**0.909**	**0.873**	**0.916**	61	19.1 ms	3.04 M	18.72G
WheatLFANet	0.905	0.843	0.900	**164**	**8.4 ms**	**0.72 M**	4.07G

Bold text indicates the best results

**Fig. 5:**
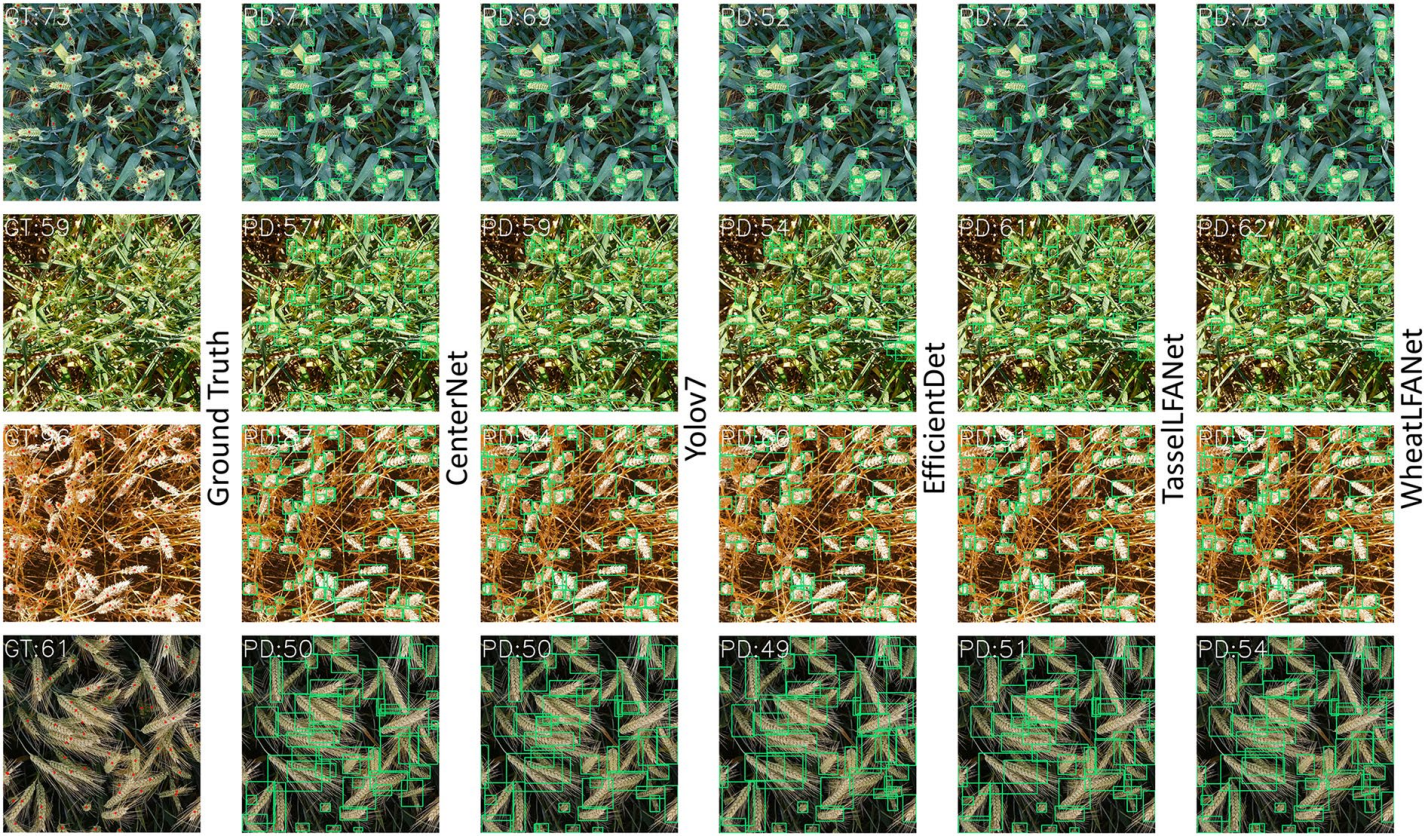
Illustration of the prediction results of different methods. GT denotes the ground-truth count and PD the predicted count. Red points are manual annotations based on the GWHD_2021 dataset.

In summary, under the condition of similar accuracy, ease of deployment and high real-time performance should be considered, especially within a wide range of resource constraints. To a certain extent, when deployed in more stringent device environments, WheatLFANet's performance will always be better than other methods because its speed exceeds other methods by an order of magnitude. Furthermore, to more intuitively demonstrate the detection performance of WheatLFANet, we provide some qualitative results in the form of example images, as shown in Fig. 5. Even in specific counting tasks, WheatLFANet maintains a strong performance level. In the majority of cases, all methods demonstrate good counting capabilities. Nonetheless, there are certain instances where EfficientDet's performance significantly declines. These differences are also related to model architecture limitations and challenges inherent in the task. From the perspective of comprehensive performance, WheatLFANet has a higher cost-effectiveness and better practicality, making it the best choice.

#### Beneficial experiments for counting tasks

**Fig. 6:**
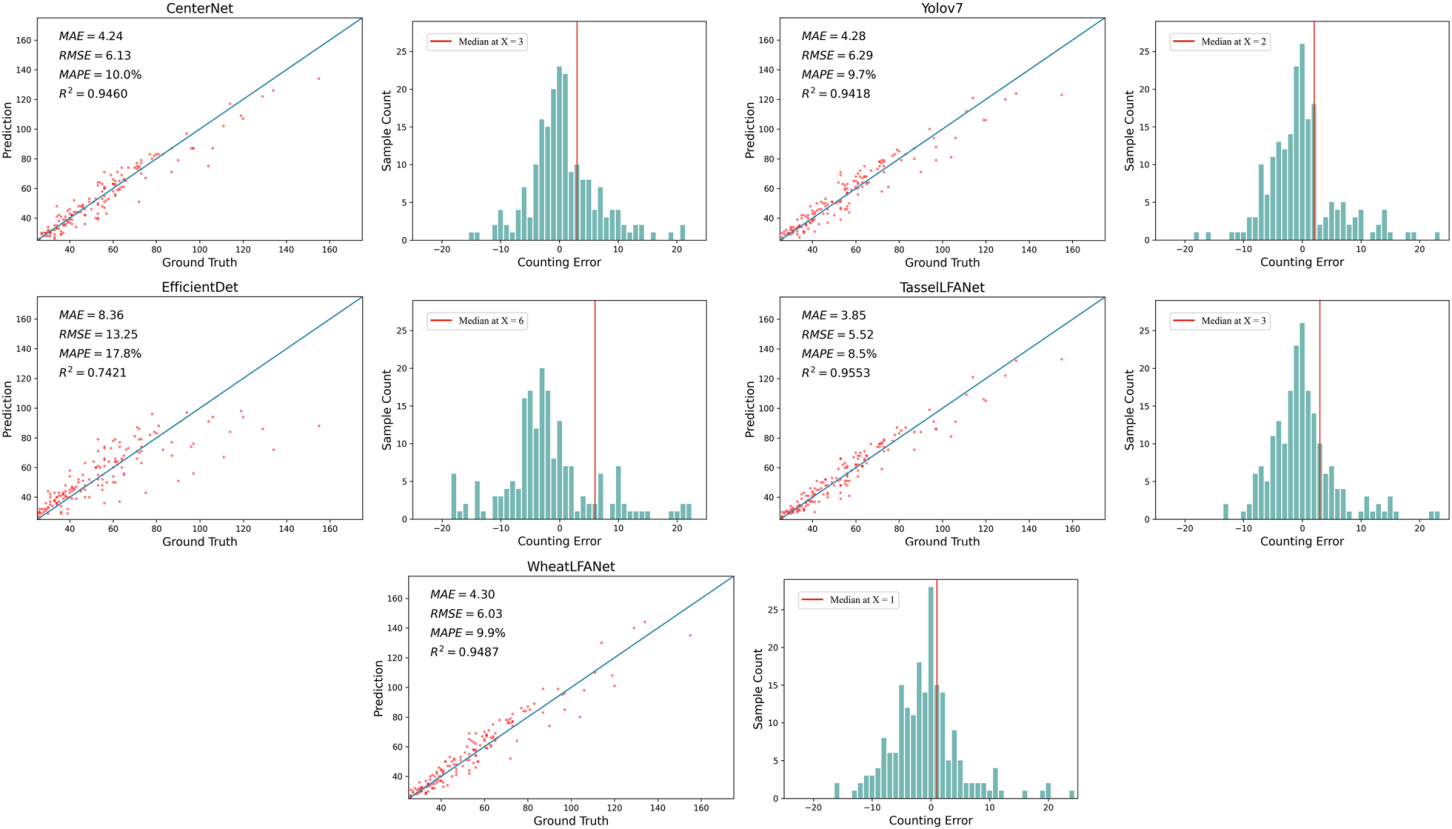
Plot of the counting test results of the models. The left figure shows the linear regression results of model predicted counts and ground-truth counts. The right figure shows the histogram of counting errors, with the median point of the counting error of each model has been marked with a red line

The importance of counting tasks is self-evident. To test the effectiveness of the models in counting tasks, we randomly selected 200 images from the predefined test dataset to further test the performance of the models in counting wheat head numbers. Among these selected images, the total number of instances counted was 9446, with an average of 47.23 instances per image. In this experiment, we plotted the linear regression and error histograms of the experimental methods and calculated the mean absolute error (MAE), root mean square error (RMSE), mean absolute percentage error (MAPE), and the coefficient of determination (R^2^). As shown in Fig. 6, we present two visual charts for each model. The left is the linear regression plot and the right is the error histogram. It can be observed that, in the scatter plot of the linear regression, all models except for EfficientDet demonstrate good fitting ability. In addition, even when the difference in the number of heads in the image becomes larger, they can still maintain good counting performance. Overall, these results are consistent with the previous experiments. Furthermore, the error histogram shows that the error distribution of the TasselLFANet and WheatLFANet models is relatively uniform, with roughly equal numbers of errors on both sides of the zero-error count point. This indicates that the two models can better capture the global information in the wheat head image, resulting in more accurate predictions. In particular, we also marked the median to better measure the location and variability of the model's counting data. The median of WheatLFANet is closest to the zero-error point, which also indicates that the hyper lightweight WheatLFANet model has better generalization and robustness, and we will provide evidence to support this later.

#### Choosing WheatLFANet:

#### The smart decision

**Fig. 7:**
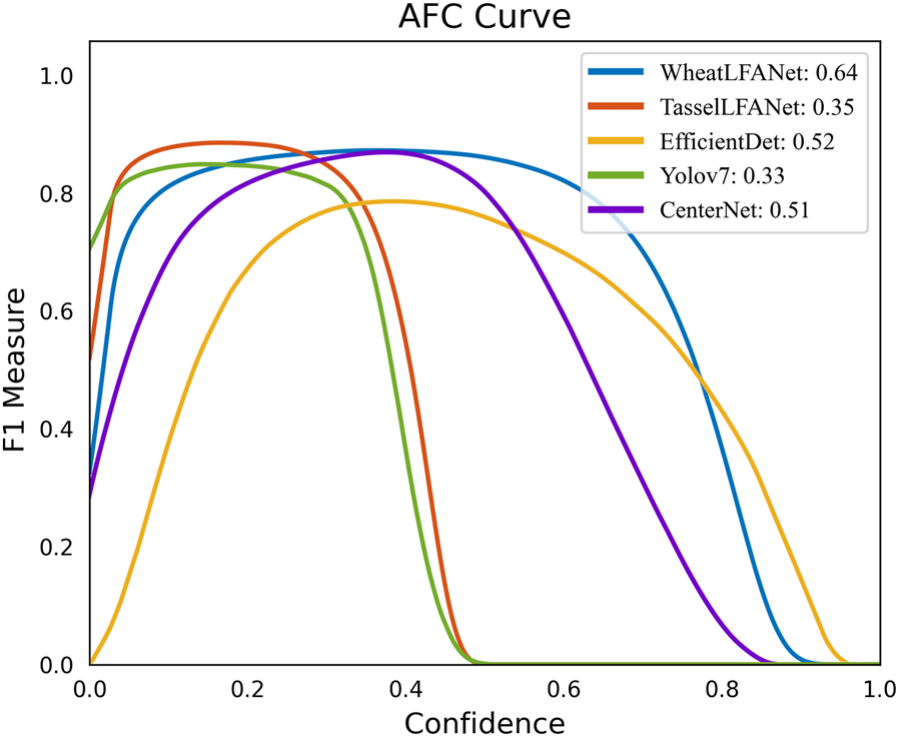
Area-F1-Confidence (AFC) Curve. The enclosing area of all curves is marked in the upper right corner

Thus far, it is believed that the reader has gained an understanding of the WheatLFANet method that has been proposed. However, we shall continue in presenting compelling evidence to substantiate that WheatLFANet represents the optimal choice. We know that accuracy and confidence are key evaluation metrics in machine learning tasks. Improving these two metrics can provide more accurate and reliable information. High confidence detection results indicate that the model has a high degree of certainty in its predictions, which can provide guidance for subsequent decision-making and actions. Therefore, we evaluated the relationship between the model's confidence and F1 measure and presented the qualitative results through visualization to make the dataset more intuitive. The plotted curve between F1 measure and confidence can reveal the changes in model accuracy and recall at different confidence levels, helping us better understand the performance of the model. As shown in Fig. 7, the model's F1 measure changes correspondingly as the confidence threshold increases from low to high. F1 is a comprehensive consideration of precision (P_r_) and recall (R_e_) used to evaluate the accuracy and completeness of the model's object detection. It is defined as:

25 $$F1 = 2\frac{{P_{r} R_{e} }}{{P_{r} + R_{e} }} \in [0,1]$$

Specifically, a higher F1 measure indicates that the model performs well in terms of both precision and recall, which means it can ensure both the accuracy and completeness of the detection results. At the same time, we labeled the area under the curve in the upper right corner of the graph. It is clear that the WheatLFANet model has the largest bounding box area, indicating that it has better generalization and robustness, and can achieve higher detection accuracy at lower confidence threshold. In comparison, TasselLFANet and Yolov7 did not perform as well as we expected. Some possible explanations are that these models suffer from issues such as error propagation and information loss during the training process [55], or their performance on detecting certain objects may not be as good as WheatLFANet. Due to their relatively high complexity, these models have more redundant features, which can lead to over-emphasizing some relevant features while neglecting other important ones [56, 57]. Moreover, there may be some hyperparameters that need to be adjusted to optimize model performance. Similar to the information presented in Figs. 3 and 7, this information can guide us in choosing the appropriate model and hyperparameters, and further improve model performance. Overall, WheatLFANet performs well in many practical aspects and is the most suitable model to use.

## Further discussions

**Fig. 8:**
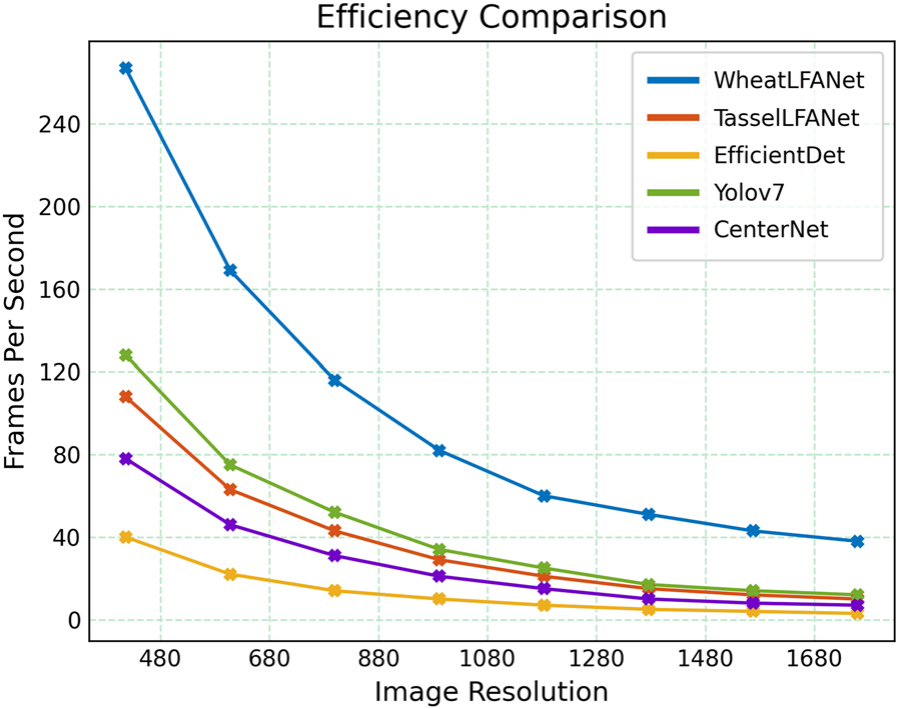
The speed at different resolutions was compared among different models, WheatLFANet emerged as the clear winner

Hard to have your cake and eat it too. In recent years, lightweight neural networks have gained attention for their efficient inference and training on resource-constrained devices. While this advantage often comes at the cost of accuracy. Therefore, while pursuing high-real-time and lightweight models, it is often difficult to balance accuracy [58–60]. Compared to the baseline model TasselLFANet, WheatLFANet achieves a significant improvement in speed by reducing the output channel size, optimizing the model architecture, and integrating various modules. However, the adjustment of output channel size inevitably decreases the model's learning capacity, resulting in a decline in accuracy. At the same time, for wheat head recognition, when all varieties of wheat heads in the GWHD_2021 dataset are covered as one category, the differences between different varieties are ignored, making it very challenging to achieve lightweight and high-real-time performance while maintaining high accuracy. From the perspective of the merged difficulty, we need to further improve the accuracy of lightweight neural networks to better play a role in wheat head detection. Some researchers have tried to improve the accuracy of the model by improving the network structure, optimizing the loss function, and introducing attention mechanisms [61–63]. Also, classifying wheat heads of different varieties is also a key factor in improving model accuracy. Therefore, we can attempt to use the multi-task learning method, enabling the model to simultaneously accomplish both wheat head detection and variety classification tasks, thereby enhancing its ability to distinguish differences between different varieties. Fortunately, the cutting-edge research of machine learning experts has provided many optimization methods and technologies for the development of lightweight neural networks, such as the combination strategy of using adaptive width diversity and depth separable convolution in MobileNetV3 [64], the local connection mode and channel attention-based cross-layer feature reuse in EfficientNetV2 [65], and the introduction of reversible network structure in network design to improve the network's representation ability in RevNet [66]. It is exciting that our implemented WheatLFANet has been able to maintain high-real-time performance on resource-limited devices, while achieving a level of performance comparable to the SOTA-performance TasselLFANet. Furthermore, as shown in Fig. 8, let’s take a look at the speed comparison between WheatLFANet and different models!

## Conclusions

In order to ensure low-latency image detection on resource-limited devices, we re-examined cutting- edge algorithms and designed a high-real-time lightweight neural network, WheatLFANet, based on TasselLFANet with improvements on the existing issues. This network can maintain ultra-high speed even on low-end devices. By effectively fusing multi- dimensional mapping of language information and cross-stage features, we achieved effective detection of diverse and complex wheat heads, with good generalization ability. This study demonstrates the feasibility of achieving ultra-real-time lightweight wheat head detection on low-end devices, and suggests that simple yet powerful neural network designs can be effective. We hope these findings will encourage more researchers to invest in detection methods in agriculture and promote further technological progress and application development.

For future work, we plan to explore more advanced deep learning algorithms such as transfer learning, reinforcement learning, and generative adversarial networks to improve the accuracy and efficiency of wheat head detection. We also consider applying these technologies to the detection of other crops, in order to further promote the development of agricultural technology and improve agricultural productivity. Overall, we hope this study will bring more technological innovation and application development to the agricultural field.

## Data Availability

The datasets used and/or analyzed during the current study available from the corresponding author on reasonable request.
